# Patient reported barriers and facilitators to using a self-management booklet for hip and knee osteoarthritis in primary care: results of a qualitative interview study

**DOI:** 10.1186/1471-2296-14-181

**Published:** 2013-12-01

**Authors:** Nienke Cuperus, Agnes J Smink, Sita MA Bierma-Zeinstra, Joost Dekker, Henk J Schers, Fijgje de Boer, Cornelia H van den Ende, Thea PM Vliet Vlieland

**Affiliations:** 1 Department of Rheumatology, Sint Maartenskliniek, PO box 9011, Nijmegen 6500, GM, The Netherlands; 2 Department of General practice, Erasmus University Medical Center, Rotterdam, The Netherlands; 3 Department of Orthopaedics, Erasmus University Medical Center, Rotterdam, The Netherlands; 4 Department of Rehabilitation Medicine, VU University Medical Center, Amsterdam, The Netherlands; 5 Department of Primary and Community Care, Radboud University Nijmegen Medical Center, Nijmegen, The Netherlands; 6 Department of Medical Humanities, VU University Medical Centre, Amsterdam, The Netherlands; 7 Department of Rheumatology, Leiden University Medical Center, Leiden, The Netherlands; 8 Department of Orthopaedics, Leiden University Medical Center, Leiden, The Netherlands

## Abstract

**Background:**

To enhance guideline-based non-surgical management of hip or knee osteoarthritis (OA), a multidisciplinary, stepped-care strategy has been implemented in primary care in a region of the Netherlands. To facilitate this implementation, the self-management booklet “Care for Osteoarthritis” was developed and introduced. The aim of the booklet was to educate patients about OA, to enhance the patient’s active role in the treatment course, and to improve the communication with health care providers. To successfully introduce the booklet on a large scale we assessed barriers and facilitators for patients to using this booklet.

**Methods:**

Seventeen primary care patients with hip or knee OA who received the self-management booklet participated in this qualitative study using semi-structured interviews. Purposive sampling was used to ensure diversity of the patients’ view about the booklet. The interviews were transcribed verbatim and analysed using a thematic analysis approach.

**Results:**

Three core themes with patient perceived barriers and facilitators to use the booklet emerged from the interviews: 1) the role of health care providers, 2) the patient’s perceptions about OA and its manageability, and 3) the patient’s perceptions about the usefulness of the booklet and patient’s information needs. Regarding the first theme, a barrier was the lack of encouragement from health care providers to use the booklet in the treatment course of OA. Moreover, patients had doubts concerning the health care providers’ endorsement of non-surgical treatment for OA. Barriers from the second theme were: thinking that OA is not treatable or that being pro-active during the treatment course is not important. In contrast, being convinced about the importance of an active participation in the treatment course was a facilitator. Third, patients’ perceptions about the usefulness of the booklet and patients’ information needs were both identified as barriers as well as facilitators for booklet use.

**Conclusions:**

This study contributes to the understanding of patient perceived barriers and facilitators to use a self-management booklet in the treatment course of OA. The results offer practical starting points to tailor the implementation activities of the booklet nationwide and to introduce comparable educational tools in OA primary care or in other chronic diseases.

## Background

Osteoarthritis (OA) is the most common joint disorder and the major cause of chronic musculoskeletal pain and disability in elderly worldwide [1]. About 18.0% of women and 9.6% of men older than 60 have symptomatic OA; its prevalence is increasing due to the increased life expectancy [2]. General practitioners (GPs) are frequently consulted by patients with hip or knee OA. The core treatment for OA, a combination of pharmacological and non-pharmacological treatment modalities such as weight management and exercise are mainly performed in primary health care [3,4]. The success of those interventions are often related to adequate self-management, therefore international guidelines stress the importance of self-management in OA [5]. Self-management refers to interventions, training, and skills with which patients with a chronic condition can learn how to effectively take care of themselves [6]. In the last decade, a growing number of studies have examined the effectiveness of self-management programs for OA, with some studies showing improvements in pain and disability [7-9].

One strategy to enhance self-management in the treatment of chronic diseases is the use of patient booklets. Based on the evidence-based, multidisciplinary, patient-centred, stepped-care strategy for hip or knee OA i.e. BART (Beating osteoARThritis) [10], a patient-friendly self-management booklet “Care for Osteoarthritis” (Zorgwijzer Artrose^©^) [11] was systematically developed in collaboration with OA patients and GPs [12] and introduced in primary health care in a region of the Netherlands. This booklet consists of three sections. The first section provides information about OA in general and gives an overview of the health care providers involved in OA care. The second section provides information about non-surgical evidence-based treatment options for hip and knee OA and its optimal sequence in three steps which is based on the stepped-care strategy for hip or knee OA. To enhance the patient’s active role in the treatment of OA as well as the communication with health care providers, the third section of the booklet contains tools to monitor symptoms, to evaluate the effect of treatment, to prepare consultations, and to formulate a comprehensive overview of the treatment options that already have been carried out. To successfully introduce this booklet on a large scale, we should evaluate its implementation among the stakeholder group of end users, i.e. the patient [13].

Several studies have demonstrated the outcome of using patient booklets in the management of a chronic disease with small improvements in physical activity [14], knowledge [15-17], and health status outcome [17-22]. These studies also showed low dissemination rates of the booklets [19,21,22], which could be an explanation for the relatively small effects that were found. Although one study examined potential barriers for the introduction of a diabetes passport using focus groups, participants in that study had never actually used the booklet [23]. To our knowledge, no studies comprehensively described why patients do or do not use such booklets in the treatment course of a chronic disease.

Previous qualitative studies have explored barriers for patients to use other non-surgical management recommendations for OA, such as physical exercise [24-28] or medication [24,28]. Commonly identified barriers that limit the patients’ use of these recommendations were patients’ perceptions about OA and its symptoms [25-27,29] and patients’ expectations regarding the benefits of non-surgical treatment for OA [25-29]. However, barriers and facilitators for patients to use a self-management booklet in OA still seems to be unknown.

The aim of this qualitative interview study was to evaluate the introduction of the booklet “Care for Osteoarthritis” by 1.) exploring how patients used the booklet and 2.) identifying patient reported barriers and facilitators to use the booklet. This knowledge can then be used as starting points to implement the booklet nationwide and to introduce comparable educational tools in OA primary care or in other chronic diseases.

## Methods

### Design

A qualitative research approach was used in order to explore patient perceived barriers and facilitators to use the booklet “Care for Osteoarthritis” [30]. We conducted semi-structured interviews [31] guided by theoretical concepts of a broad health-related behaviour model; i.e. the Integrated Change (I-Change) model [32]. This qualitative approach offers patients the opportunity to present their views in their own words about the use of the booklet and allow them to address themes of which the researchers might not be aware of. Written informed consent from participants to record the interview was obtained prior to the start of the interview. The Institutional Review Board of the University Medical Centre, Nijmegen approved the study (protocol number: 2012/133).

### Participants

We invited a sample of patients who had participated in an umbrella project that aims to implement the stepped-care strategy for hip and knee OA in primary care in a region of the Netherlands and to evaluate the implementation process (i.e. the BART-project) [10]. In this broader project executed from August 2010 to March 2013, a cohort of 313 patients who visited their primary care general practices with a new episode of hip or knee complaints due to (symptomatic) hip or knee OA were included by their GP. To implement the stepped-care strategy, several implementation activities aligned to patients as well as different health care providers were developed, performed and evaluated in the BART-project [33,34]. Patients received the booklet “Care for Osteoarthritis” from their GP or from one of the researchers and were instructed on how to use it. Every six months for two years quantitative data were collected by patient-reported questionnaires to identify the patient’s health seeking behaviour and health status. Considering the large individual variation in the course of OA symptoms and the likelihood that the patient would consult the booklet during a period characterized by complaints, we approached the participants for this qualitative study 12 months after they had received the booklet. To ensure diversity of the patients’ view about the booklet, purposive sampling was used to identify potential participants. We randomly selected participants based on their answer to the question included in the questionnaire of the BART-project one year after baseline: “Do you use the booklet “Care for Osteoarthritis”?” (yes, regularly/yes, occasionally/yes, but not in the past six months/no, never). Except the category “yes, regularly”, we selected patients in blocks from all the other categories. We were not able to include patients in the category “yes, regularly” as only one patient selected that answer in the questionnaire but refused to participate in this qualitative study.

### Data collection

Data collection took place between 12 and 18 months after distributing the booklet through semi-structured interviews at the participants’ homes. Two female researchers (AS, NC) working at the rheumatology research department of a specialized hospital in the Netherlands conducted the interviews. The equipment we used to record the interviews provided both audio and video tapes. However, before the start of the data analysis we decided for pragmatic reasons to use only the audio tapes. Alternately, one of the researchers guided the interview and asked the questions, while the other researcher made field notes. In two cases, the partner of the participant was present during the interview. Both researchers were also responsible for the data collection in the BART-project; no other relationship existed with the participants prior to the interviews.

An interview guide with open-ended questions was developed (Table 1) with topics that were derived from a broad behavioural model i.e. the I-Change model. This model postulates that behaviour is the result of a person’s intention which is in turn influenced by four factors: motivational factors (e.g. attitudes, social influences, efficacy), awareness factors (e.g. knowledge, risk perceptions, cues to action), information factors (e.g. quality of messages or sources used) and predisposing factors (e.g. personality, environment) [32]. We selected the I-Change model as the theoretical framework for the current study as we aimed to identify a broad spectrum of barriers and facilitators for patients to use the booklet. Since the I-Change model incorporates insights of several behavioural models, its integrated nature made it feasible to explore a broad spectrum of potential barriers and facilitators. The interview guide was structured around four constructs of the I-Change model: behaviour (i.e. booklet use), motivation factors (i.e. attitude towards the booklet and OA, efficacy to use the booklet), awareness factors (knowledge about OA, severity of OA, self-management) and information factors (i.e. information received from health care providers). In addition, we were interested if patients had suggestions for improvement of the booklet. We did not ask patients about their current intention with regard to using the booklet as patients were interviewed 12 months after they had received the booklet; predisposing factors were already assessed by the questionnaires of the BART-project. The co-authors reviewed the questions for both content and format. The use of an interview guide ensured that the main issues related to the model would be discussed. The questions had an open-ended format to provide patients with the scope to talk about their experiences and perspectives freely and in their own words. Three pilot interviews were held, which led to an adjustment in the wording of the questions. The number of interviews performed was determined by consensual agreement of the researchers that analytical saturation had been achieved i.e. the coding process (as described below) revealed no new information [35].

**Table 1:** Interview guide

	
Behaviour	Do you use the booklet?
	How do you use the booklet?
Motivation	Why do you use (or not use) the booklet?
	Are you interested in information about OA and the treatment options?
	What do you think about the booklet?
Awareness	What do you know about OA and the treatment options?
	How severe are your symptoms in daily life?
	What can you do to influence OA symptoms?
Information	What did your health care provider tell you about the booklet when you received it?
	Did you ever discuss the booklet with a health care provider?
	What did health care providers tell you about OA?
Suggestion for improvement	Do you have any suggestions to improve the booklet?

### Data analysis

To analyse the data, a thematic analysis approach was used in order to systematically organize the data and then to identify repeated patterns (themes) across the data with regard to the research question [36]. First, each interview was transcribed verbatim to facilitate transparency [37]. The interview transcripts were carefully read by the researchers who also conducted the interviews to ensure validity of the transcripts. Subsequently fragments of meaning within the text in relation to the research question were coded after each interview had taken place. To increase the reliability of the coding process triangulation of researchers was used: both researchers independently coded the interviews. Afterwards, the two researchers compared, discussed, and, if necessary, adjusted their coding. All interviews were analysed with support of the qualitative analysis software program MAXQDA® 10 (VERBI software GmBH, Germany) to order the data. Further analysis was conducted after all the interviews were coded. The two researchers grouped similar codes together into sub-themes, which in turn were organized and clustered together into core themes. As the analysis progressed, the constant comparison and review of the data yielded a number of core themes and sub-themes. During this iterative process, these core themes became the basis for the exploration of barriers and facilitators in the patients’ use of the booklet. Peer debriefing was used: the emerging themes were discussed with the co-authors and an expert in qualitative research (FdB).

## Results

### Participants

Twenty-six patients were approached by telephone to participate in the study. Of those, nine refused to participate because they either did not believe that they could give meaningful information that would contribute to the research question (n = 5) or were not comfortable with being interviewed (n = 4). Five of these nine non-participants had reported in the questionnaire of the BART-project that they had never used the booklet. Interviews were conducted with 17 patients, of whom 12 were women and 5 men, with a median age of 67 years (52-85) (Table 2). Median Western Ontario and McMaster Universities Arthritis Index (WOMAC) score was 77 (55-94). The median interview duration was 35 minutes (20-58). Due to a technical defect, the audio and video tapes of one interview were missing; field notes of this interview were used for analysis. All other data were complete.

**Table 2:** Characteristics of the 17 participants and their booklet use

**Participant number**	**Gender**	**Age**	**Duration interview (minutes)**	**Duration of complaints (years)**	**OA severity (WOMAC)**^**1**^	**Booklet use questionnaire**^**2**^	**Booklet use interview**^**3**^
1	F	71	51	>10	71	Y^nr^	R
2	F	85	?	<1	73	Y^nr^	U
3	M	67	33	1-5	90	Y^nr^	R
4	M	67	47	5-10	73	N	N
5	M	52	30	>10	71	Y^o^	N
6	F	68	45	1-5	82	N	N
7	F	63	25	1-5	71	N	N
8	M	65	58	1-5	84	Y^o^	U
9	F	69	25	>10	79	N	R
10	F	59	22	<1	61	Y^o^	R
11	F	67	24	1-5	94	Y^nr^	U
12	F	60	39	5-10	64	Y^o^	U
13	M	71	55	5-10	55	Y^o^	R
14	F	59	37	>10	89	N	R
15	F	71	58	5-10	79	Y^o^	R
16	F	76	20	<1	82	Y^o^	N
17	F	70	23	1-5	77	N	R

^1^standardized WOMAC scores (from questionnaire). Higher scores indicate worse pain, stiffness, and functional limitations.

^2^categorization based on answers given in the questionnaire.

^3^categorization based on answers given in the interview.

*Abbreviations:F* female, *M* male, *?* data missing, *Y*^*nr*^ Yes, but not recently, *Y*^*o*^ Yes, occasionally, *N* Not ever, *U* User, *R* Reader, *N* Non-user.

### Booklet use

The data analysis showed that the booklet was used in different manners: some patients only read the booklet while others used the self-management tools in the third section of the booklet. Based on the interviews, we categorized patients according to their booklet use; we determined which patients were actual ‘users’ (i.e. made notes in the booklet, brought the booklet to the consultation with a health care provider, or used the tools), which ‘readers’ (i.e. only read the booklet), and which ‘non-users’ (i.e. neither read nor used the booklet). The answers presented below illustrate the difference in how users, readers and non-users responded to the questions regarding their booklet use.

8 (u): “*For me the booklet serves as a mnemonic device. When I read the booklet, I mark important information and discuss this with the GP during a consultation.”*

10 (r): *“I only read the booklet once and then put it away. After that I have never used the booklet.”*

6 (n): *“I never read the booklet.”*

According to the abovementioned categorization, the study population included four users, eight readers, and five non-users. Patients’ answer to the question “do you use the booklet” varied across the method of administration i.e. questionnaire or interview (Table 2). For the categorization of booklet use we decided to use all the information gathered by the interviews, rather than the information obtained from the self-report questionnaires of the umbrella project, because in the interviews patients received room to explain and clarify the utilization of the booklet in more detail.

### Barriers and facilitators for booklet use

Based on the interviews several patient perceived barriers and facilitators to use the self-management booklet in OA care emerged from the data analysis (Table 3). These barriers and facilitators can be divided into three core themes: 1) the role of health care providers, 2) the patient’s perceptions about OA and its manageability and 3) the patient’s perceptions about the usefulness of the booklet and patient’s information needs.

**Table 3:** Overview of the patient reported barriers and facilitators to use the self-management booklet

	
**Theme 1: role of health care providers**	
***Barriers***	Lack of clear information about how to use the booklet given by the health care providers
	Lack of encouragement from health care providers to use the booklet in the treatment course of OA
	Patients’ doubts concerning the health care providers’ endorsement of non-surgical treatment modalities for OA
***Facilitator***	Encouragement from health care providers to use the booklet in the treatment course of OA
**Theme 2: patient’s perceptions about OA and its manageability**	
***Barriers***	Patients’ perceptions of OA as inevitable or not curable
	Patients’ perceptions that the complaints due to OA are not severe enough
	Thinking that being pro-active during the treatment course is not an effective strategy to control the disease course
***Facilitator***	Being convinced of the importance of an active participation in the treatment course of OA
**Theme 3: patient’s perceptions about the usefulness of the booklet and patient's information needs**	
***Barriers***	Patients’ perceptions that the booklet is not a useful tool to manage their OA or not being aware of the aims of the booklet
	Having already sufficient knowledge about OA or sufficient support from health care providers
	Not willing to know everything about OA or not paying any attention to OA
***Facilitators***	Patients’ perceptions that the booklet is a useful tool to manage their OA
	Lack of knowledge about OA or being interested in having more information

### Theme 1: the role of health care providers

We identified three barriers and a facilitator in booklet use related to health care providers. First, the majority of patients reported that they had not received any clear information about how to use the booklet from their health care providers. Among those who had been informed to read the booklet, some reported that while having been advised to read the booklet, no instructions were given about how to use it.

7 (n): *“The GP did not give me any information about the booklet when I received it.”*

13 (r): *“When I received the booklet, the GP only told me to have a look inside.”*

Despite the limited information patients had received about the booklet, this did not impede some patients from using the booklet.

11 (u): *“The GP did not explain how to use the booklet, I figured it out by myself.”*

The facilitator for booklet use seems to be the encouragement of health care providers to use the booklet in the management of OA. One patient reported to be encouraged by the GP to use the booklet to monitor symptoms and to discuss this during their consultation.

2 (u): *“I take the booklet every time I visit the GP. Together with the GP I fill in the third section of the booklet, I monitor my symptoms by using the booklet and I discuss this with the GP.”*

None of the other patients reported that they have used the booklet during a consultation after receiving it; in short they were neither encouraged to monitor their symptoms using the booklet nor asked to bring it to subsequent consultations. One patient even suggested that the GP actually discouraged the use of the booklet.

12 (u): *“When I tried to discuss the content of the booklet with my GP, he told me that the booklet is redundant as they have all the information in the computer.”*

Patients reported the limited time during a consultation as a reason for the lack of information and the limited encouragement they received from their health care providers to use the booklet.

4 (n): *“The booklet was never discussed with the GP, they do not have time for that, you only have ten minutes.”*

Thirdly, patients perceived doubts concerning the health care providers’ endorsement of non-surgical treatment modalities for OA what might be a barrier for booklet use. Such doubts were reported by the ’non-users’ in particular.

13 (r): *“The GP told me that a total knee replacement was the only option for me. I asked him about physical therapy, but he told me it would not be useful for me.”*

6 (n): *“The GP told me that it will only get worse, instead of better.”*

16 (n): *“The orthopaedic surgeon told me that there is nothing we can do.”*

### Theme 2: the patient’s perceptions about OA and its manageability

Analysis of the interviews showed that whether or not patients used the booklet might have been influenced by their own perceptions about OA and how to manage it. Barriers to use the booklet were patients’ perceptions that OA is not treatable, that their complaints were not severe enough, or that being pro-active during the treatment course is not important.

15 (r): *“In my opinion, there is nothing to do about OA. Therefore I do not need any advice.”*

14 (r): *“For me, the complaints are not very serious. I am doing fine.”*

4 (n): *“I cannot change anything about OA. The GP is the expert. He knows what to do”.*

On the other hand, a patient’s perception about OA can also be a facilitator: some perceived that an active participation was important in the treatment course of OA and therefore used the booklet.

8 (u): *“I think I can control the symptoms myself. In my opinion, it is important to know what to do, instead of waiting until it gets worse and afterwards regretting that I should have acted earlier.”*

11 (u): *“I think I can minimize the symptoms caused by OA by managing it myself.”*

In addition, the patients’ concern about the prognosis of OA seems another facilitator to use the booklet.

8 (u): *“I think the symptoms will deteriorate rather than improve. I hope it will not get worse and I shall make every effort to do so.”*

After having studied the perceptions about OA among the three patient groups, some differences can be addressed. Non-users and readers were less positive with respect to the extent to which OA is treatable and considered active participation in their own treatment course less important, whereas users perceived that active participation is indeed important in the management of OA.

### Theme 3: the patient’s perceptions about the usefulness of the booklet and patient’s information needs

This theme refers to patient perceptions about whether the booklet can be a useful tool in the management of OA as well as to patient information needs; both could serve as either barriers or facilitators. Some patients believed that the booklet is not a useful tool or were not aware of the aims of the booklet. As a consequence, these patients did not read or use the booklet.

6 (n): *“I have not read the booklet. In my opinion the booklet is not useful as it will not change anything about my complaints.”*

In contrast, patient’s perceptions about the usefulness of the booklet might also be a facilitator as some patients thought that the booklet provided the opportunity to make a comprehensive overview of the treatment options that already have been tried (referring to the third section of the booklet) or that it makes information accessible.

1 (r): *“This tool creates a clear overview of all received treatment options for all health care providers.*

8 (u): *“An advantage of the booklet is that you can read it as many times you want. You have all the time to read it. I already have read it three or four times.”*

The second barrier and facilitator are the patient’s information needs. Patients were not willing to seek information as they believed that they already knew the information found in the booklet, did not want to know everything about OA, did not pay any attention to their OA, or felt to be sufficiently supported by their health care providers.

4 (n): “*I definitely do not want to know everything about my disease.”*

17 (r): *“The booklet is not an appropriate tool for me, I ignore having OA. Only if you are convinced of really having OA, then the booklet might be useful.”*

9 (r): “*I do not use the booklet, as I have adequate support from my GP, physical therapist, and physician assistant.”*

On the other hand, information needs of the patient also serve as facilitators: some patients believed that their knowledge about OA was insufficient or were interested in having more information and, therefore, read or used the booklet.

8 (u): *“I had little knowledge about OA. Therefore, I considered it important to learn more.”*

In this theme differences between users, readers and non-users could also be addressed. In particular, the non-users did not perceive the booklet to be very useful, whereas readers or users did. Some readers and non-users were not willing to seek information whereas users were interested in more information.

## Discussion

To our knowledge, this is the first qualitative study that provides insight into patient perceived barriers and facilitators to use a self-management booklet in the treatment course of OA. Three core themes with barriers and facilitators emerged from the interviews. Regarding the first theme, a barrier to use the booklet was the lack of encouragement from health care providers to use the booklet in the treatment course of OA. Moreover, patients had doubts concerning the health care providers’ endorsement of non-surgical treatment for OA. In contrast, encouragement from health care providers to use the booklet was a facilitator. Barriers from the second theme were: thinking that OA is not treatable or that being pro-active during the treatment course is not important, whereas being convinced of the importance of an active participation was a facilitator. Third, patients’ perceptions about the usefulness of the booklet and patients’ information needs were both identified as barriers as well as facilitators for booklet use.

Before discussing the results, some limitations need to be addressed. First, it is important to recognize that the patients’ perceptions might not coincide with their own or their health care providers actual behaviour. We did not cover the health care providers’ perceptions about the booklet because this study was restricted to patients, while barriers and facilitators can also act at other levels of the health care system [13]. Second, we did not include patients who reported to use the booklet regularly in the questionnaire of the BART-project. During the current study, only one patient reported to use the booklet regularly but refused to participate as the patient believed not being able of giving meaningful information. Moreover, the patients answers to the question “do you use the booklet” were inconsistent between the questionnaires and interviews. We categorized booklet use based on the interviews as we considered these answers most valid because in the interviews we were able to explore in more detail how patients used the booklet. Although this could have resulted in missing relevant barriers or facilitators, we believed this was not very likely as the data collection was continued until analytical saturation was achieved. Another limitation might be recall bias, particularly in the non-users as patients were interviewed 12 to 18 months after receiving the booklet. Finally, the identified themes present some of the reasons for patients with OA to make use of a self-management booklet, other themes could emerge due to differences in ethnic background, culture or health care systems.

According to the interviewed patients, they received little encouragement from their health care providers to use the booklet: patients reported that health care providers gave no or few instructions regarding how to use the booklet, did not encourage booklet use, and did not refer to it in subsequent consultations. This finding is in line with a study examining booklet use in hypertensive patients; only 10% of these patients were asked by their GP about the booklet after receiving it [22]. Patients in our study perceived lack of consultation time as an explanation for the limited encouragement they received; probably practice nurses could be involved [38]. Another explanation might be that patients have low expectations regarding the GP’s encouragement of the booklet what prevented patients from discussing the booklet as has been shown in a study on the implementation of a diabetes passport [23]. Nonetheless, our results imply that patients need information and encouragement from their health care providers to use the booklet in the management of OA.

Interestingly, patients in our study reported doubts concerning the health care providers’ endorsement of non-surgical treatment modalities for OA. It has been shown in previous qualitative studies that GPs and rheumatologists consider existing non-surgical treatments insufficiently effective, with a total knee replacement as ultimate and only efficient treatment option [24]. Rosemann et al. [39] showed that GPs hardly tried to motivate patients to change their behaviour to effectively manage OA, as GPs considered its success rate too low. The doubts of patients in our study concerning the health care providers’ views of non-surgical treatment for OA might have hindered patients to use the booklet as the health care provider’s attitudes and beliefs can affect the patients’ perceptions [40] and preferences for treatment [41,42]. Recently it has been shown that attitudes of health care providers remain a major barrier for patients to be more actively involved in their treatment course [43]. A more explicitly expressed positive attitude of health care providers towards the benefits of non-surgical treatment for OA might encourage patients to use the booklet.

Patient’s reluctance to use the booklet might have been caused by the patient’s perceptions of OA as inevitable or not curable, a barrier also found in studies examining the patients’ use of other non-surgical OA recommendations [24,27,28]. Some patients in our study perceived that being pro-active is not an effective strategy to control the disease course. In a study on illness perceptions it has been stated that patients create their own beliefs about whether the illness is controllable or curable, which determines self-management behaviour [44]. Considering this, health care providers should explore the patient’s illness perceptions before introducing the booklet and if considered inadequate, the perceptions should be discussed in order to improve booklet use. However, it should be noted that, despite additional guidance, the booklet might not be an appropriate tool for every patient. For example, some patients tend to leave the control of their disease to powerful others (the doctor knows best) and do not want to be involved in making decisions regarding their treatment [43,45-47]; these patients might benefit from a more direct approach such as verbal instruction.

The results suggest that some patients did not perceive the booklet as a useful tool to manage OA and therefore did not use it. Perhaps the lack of instructions given in the booklet itself is a contributing factor. Besides, the need for information varied among patients. Some patients were not interested in reading or actively searching for information. These findings are supported in studies on factors influencing patient’s reading and seeking of written information, showing that the patients’ lack of interest in seeking information was associated with their coping styles [45,46]. For example, some patients search for all kinds of information, whereas others avoid information [48]. Therefore, health care providers need to move from a ‘one size fits all’ method of providing information to a more patient specific approach that considers the unique needs of each patient.

## Conclusions

Given the above mentioned findings, patients need encouragement from their health care providers to use the self-management booklet in the treatment course of OA, as patients legitimise non-use of the booklet by the lack of encouragement given by their health care providers and by their perceived doubts concerning the health care providers’ endorsement of non-surgical treatment for OA. Moreover, patients’ illness perceptions, perceptions about the usefulness of the booklet and patients’ information needs are important factors in booklet use. This study contributes to the field of primary health care by understanding barriers and facilitators for patients to use a self-management booklet in the treatment course of OA. The results offer starting points to tailor the implementation activities of the booklet “Care for Osteoarthritis” on a large scale or to introduce comparable tools in OA primary care or in other chronic diseases.

## Competing interests

The authors declare that they have no competing interests.

## Authors’ contributions

All authors participated in the design of the study. AJS and NC carried out the data collection. AJS, NC, CHME, TPMVV and FB were responsible for the analysis and interpretation of the data. AJS and NC were responsible for drafting the article, all other authors critical reviewed the article. Furthermore, all authors approved the final version of the article.

## Pre-publication history

The pre-publication history for this paper can be accessed here:

http://www.biomedcentral.com/1471-2296/14/181/prepub

## References

[B1] Arden N Nevitt MC Osteoarthritis: epidemiology Best Pract Res Clin Rheumatol 2006 20 3 25 10.1016/j.berh.2005.09.007 16483904

[B2] World Health Organization The global burden of disease: 2004 update 2004 Geneva: World Health Organization

[B3] Fernandes L Hagen KB Bijlsma JW Andreassen O Christensen P Conaghan PG Doherty M Geenen R Hammond A Kjeken I Lohmander LS Lund H Mallen CD Nava T Oliver S Pavelka K Pitsillidou I da Silva JA de la Torre J Zanoli G Vliet Vlieland TP EULAR recommendations for the non-pharmacological core management of hip and knee osteoarthritis Ann Rheum Dis 2013 72 1125 1135 10.1136/annrheumdis-2012-202745 23595142

[B4] Zhang W Moskowitz RW Nuki G Abramson S Altman RD Arden N Bierma-Zeinstra S Brandt KD Croft P Doherty M Dougados M Hochberg M Hunter DJ Kwoh K Lohmander LS Tugwell P OARSI recommendations for the management of hip and knee osteoarthritis, Part II: OARSI evidence-based, expert consensus guidelines Osteoarthritis Cartilage 2008 16 137 162 10.1016/j.joca.2007.12.013 18279766

[B5] Hochberg MC Altman RD April KT Benkhalti M Guyatt G McGowan J Towheed T Welch V Wells G Tugwell P American College of Rheumatology 2012 recommendations for the use of nonpharmacologic and pharmacologic therapies in osteoarthritis of the hand, hip, and knee Arthritis Care Res (Hoboken) 2012 64 465 474 22563589 10.1002/acr.21596

[B6] Barlow J Wright C Sheasby J Turner A Hainsworth J Self-management approaches for people with chronic conditions: a review Patient Educ Couns 2002 48 177 187 10.1016/S0738-3991(02)00032-0 12401421

[B7] Coleman S Briffa NK Carroll G Inderjeeth C Cook N McQuade J A randomised controlled trial of a self-management education program for osteoarthritis of the knee delivered by health care professionals Arthritis Res Ther 2012 14 R21 10.1186/ar3703 22284848 PMC3392814

[B8] Heuts PH de Bie R Drietelaar M Aretz K Hopman-Rock M Bastiaenen CH Metsemakers JF van Weel C van Schayck O Self-management in osteoarthritis of hip or knee: a randomized clinical trial in a primary healthcare setting J Rheumatol 2005 32 543 549 15742451

[B9] Warsi A LaValley MP Wang PS Avorn J Solomon DH Arthritis self-management education programs: a meta-analysis of the effect on pain and disability Arthritis Rheum 2003 48 2207 2213 10.1002/art.11210 12905474

[B10] Smink AJ van den Ende CH Vliet Vlieland TP Swierstra BA Kortland JH Bijlsma JW Voorn TB Schers HJ Bierma-Zeinstra SM Dekker J "Beating osteoARThritis": development of a stepped care strategy to optimize utilization and timing of non-surgical treatment modalities for patients with hip or knee osteoarthritis Clin Rheumatol 2011 30 1623 1629 10.1007/s10067-011-1835-x 21887488

[B11] Smink AJ Care booklet "Care for Osteoarthritis" (in Dutch: "Zorgwijzer Artrose©"). Bone& Joint Decade NL 2013 Available at http://www.artrosezorgnet.nl/diagnose-behandeling/ZorgwijzerArtrose/

[B12] Francis N Wood F Simpson S Hood K Butler CC Developing an 'interactive' booklet on respiratory tract infections in children for use in primary care consultations Patient Educ Couns 2008 73 286 293 10.1016/j.pec.2008.07.020 18723306

[B13] Grol R Grimshaw J From best evidence to best practice: effective implementation of change in patients' care Lancet 2003 362 1225 1230 10.1016/S0140-6736(03)14546-1 14568747

[B14] Williams NH Amoakwa E Belcher J Edwards RT Hassani H Hendry M Burton K Lewis R Hood K Jones J Bennett P Linck P Neal RD Wilkinson C Activity Increase Despite Arthritis (AIDA): phase II randomised controlled trial of an active management booklet for hip and knee osteoarthritis in primary care Br J Gen Pract 2011 61 452 458 10.3399/bjgp11X588411 PMC3145528 21801537

[B15] Barlow JH Wright CC Knowledge in patients with rheumatoid arthritis: a longer term follow-up of a randomized controlled study of patient education leaflets Br J Rheumatol 1998 37 373 376 10.1093/rheumatology/37.4.373 9619885

[B16] Dawes MG Kaczorowski J Swanson G Hickey J Karwalajtys T The effect of a patient education booklet and BP 'tracker' on knowledge about hypertension. A randomized controlled trial Fam Pract 2010 27 472 478 10.1093/fampra/cmq048 20631056

[B17] Maggs FM Jubb RW Kemm JR Single-blind randomized controlled trial of an educational booklet for patients with chronic arthritis Br J Rheumatol 1996 35 775 777 10.1093/rheumatology/35.8.775 8761192

[B18] Dijkstra R Braspenning J Grol R Implementing diabetes passports to focus practice reorganization on improving diabetes care Int J Qual Health Care 2008 20 72 77 18024508 10.1093/intqhc/mzm051

[B19] Dijkstra RF Braspenning JC Huijsmans Z Akkermans RP van Ballegooie E ten Have P Casparie T Grol RP Introduction of diabetes passports involving both patients and professionals to improve hospital outpatient diabetes care Diabetes Res Clin Pract 2005 68 126 134 10.1016/j.diabres.2004.09.020 15860240

[B20] Simmons D Gamble GD Foote S Cole DR Coster G The New Zealand diabetes passport study: a randomized controlled trial of the impact of a diabetes passport on risk factors for diabetes-related complications Diabet Med 2004 21 214 217 10.1111/j.1464-5491.2004.01047.x 15008829

[B21] Warner JP King M Blizard R McClenahan Z Tang S Patient-held shared care records for individuals with mental illness. Randomised controlled evaluation Br J Psychiatr 2000 177 319 324 10.1192/bjp.177.4.319 11116772

[B22] Watkins CJ Papacosta AO Chinn S Martin J A randomized controlled trial of an information booklet for hypertensive patients in general practice J R Coll Gen Pract 1987 37 548 550 3503941 PMC1711180

[B23] Dijkstra R Braspenning J Grol R Empowering patients: how to implement a diabetes passport in hospital care Patient Educ Couns 2002 47 173 177 10.1016/S0738-3991(01)00196-3 12191541

[B24] Alami S Boutron I Desjeux D Hirschhorn M Meric G Rannou F Poiraudeau S Patients' and practitioners' views of knee osteoarthritis and its management: a qualitative interview study PLoS One 2011 6 19634 10.1371/journal.pone.0019634 PMC3088707 21573185

[B25] Campbell R Evans M Tucker M Quilty B Dieppe P Donovan JL Why don't patients do their exercises? Understanding non-compliance with physiotherapy in patients with osteoarthritis of the knee J Epidemiol Community Health 2001 55 132 138 10.1136/jech.55.2.132 11154253 PMC1731838

[B26] Hendry M Williams NH Markland D Wilkinson C Maddison P Why should we exercise when our knees hurt? A qualitative study of primary care patients with osteoarthritis of the knee Fam Pract 2006 23 558 567 10.1093/fampra/cml022 16731544

[B27] Petursdottir U Arnadottir SA Halldorsdottir S Facilitators and barriers to exercising among people with osteoarthritis: a phenomenological study Phys Ther 2010 90 1014 1025 10.2522/ptj.20090217 20466741

[B28] Poitras S Rossignol M Avouac J Avouac B Cedraschi C Nordin M Rousseaux C Rozenberg S Savarieau B Thoumie P Valat JP Vignon E Hilliquin P Management recommendations for knee osteoarthritis: how usable are they? Joint Bone Spine 2010 77 458 465 10.1016/j.jbspin.2010.08.001 20851659

[B29] Carr A Barriers to the effectiveness of any intervention in OA Best Pract Res Clin Rheumatol 2001 15 645 656 10.1053/berh.2001.0179 11567545

[B30] Pope C Mays N Reaching the parts other methods cannot reach: an introduction to qualitative methods in health and health services research BMJ 1995 311 42 45 10.1136/bmj.311.6996.42 7613329 PMC2550091

[B31] Britten N Qualitative interviews in medical research BMJ 1995 311 251 253 10.1136/bmj.311.6999.251 7627048 PMC2550292

[B32] de Vries H Mesters I van de Steeg H Honing C The general public's information needs and perceptions regarding hereditary cancer: an application of the Integrated Change Model Patient Educ Couns 2005 56 154 165 10.1016/j.pec.2004.01.002 15653244

[B33] Smink AJ Bierma-Zeinstra SM Dekker J Vliet Vlieland TP Bijlsma JW Swierstra BA Kortland JH Voorn TB van den Ende CH Schers HJ Agreement of general practitioners with the guideline-based stepped-care strategy for patients with osteoarthritis of the hip or knee: a cross-sectional study BMC Fam Pract 2013 14 33 10.1186/1471-2296-14-33 23497253 PMC3602050

[B34] Smink AJ Dekker J Vliet Vlieland TP Swierstra BA Kortland JH Bijlsma JW Teerenstra S Voorn TB Bierma-Zeinstra SM Schers HJ van den Ende CH Health care use of patients with osteoarthritis of the hip or knee after implementation of a stepped care strategy Arthritis Care Res (Hoboken) doi: 10.1002/acr.22222, in press 10.1002/acr.22222 25200737

[B35] Tong A Sainsbury P Craig J Consolidated criteria for reporting qualitative research (COREQ): a 32-item checklist for interviews and focus groups Int J Qual Health Care 2007 19 349 357 10.1093/intqhc/mzm042 17872937

[B36] Braun V Clarke V Using thematic analysis in psychology Qual Res Psychol 2006 3 77 101 10.1191/1478088706qp063oa

[B37] Fossey E Harvey C McDermott F Davidson L Understanding and evaluating qualitative research Aust N Z J Psychiatry 2002 36 717 732 10.1046/j.1440-1614.2002.01100.x 12406114

[B38] van Eijk-Hustings Y van Tubergen A Bostrom C Braychenko E Buss B Felix J Firth J Hammond A Harston B Hernandez C Huzjak M Korandova J Kukkurainen ML Landewe R Mezieres M Milincovic M Moretti A Oliver S Primdahl J Scholte-Voshaar M Torre-Aboki J Waite-Jones J Westhovens R Zangi HA Heiberg T Hill J EULAR recommendations for the role of the nurse in the management of chronic inflammatory arthritis Ann Rheum Dis 2012 71 13 19 10.1136/annrheumdis-2011-200185 22039168

[B39] Rosemann T Wensing M Joest K Backenstrass M Mahler C Szecsenyi J Problems and needs for improving primary care of osteoarthritis patients: the views of patients, general practitioners and practice nurses BMC Musculoskelet Disord 2006 7 48 10.1186/1471-2474-7-48 16749935 PMC1524764

[B40] Holden MA Nicholls EE Young J Hay EM Foster NE UK-based physical therapists' attitudes and beliefs regarding exercise and knee osteoarthritis: findings from a mixed-methods study Arthritis Rheum 2009 61 1511 1521 10.1002/art.24829 19877105

[B41] Blakeman T Macdonald W Bower P Gately C Chew-Graham C A qualitative study of GPs' attitudes to self-management of chronic disease Br J Gen Pract 2006 56 407 414 16762121 PMC1839014

[B42] Carnes D Anwer Y Underwood M Harding G Parsons S Influences on older people's decision making regarding choice of topical or oral NSAIDs for knee pain: qualitative study BMJ 2008 336 142 145 10.1136/bmj.39401.699063.BE 18056742 PMC2206263

[B43] Frosch DL May SG Rendle KA Tietbohl C Elwyn G Authoritarian physicians and patients' fear of being labeled 'difficult' among key obstacles to shared decision making Health Aff (Millwood) 2012 31 1030 1038 10.1377/hlthaff.2011.0576 22566443

[B44] Kaptein AA Klok T Moss-Morris R Brand PL Illness perceptions: impact on self-management and control in asthma Curr Opin Allergy Clin Immunol 2010 10 194 199 10.1097/ACI.0b013e32833950c1 20386435

[B45] Ek S Heinstrom J Monitoring or avoiding health information–the relation to inner inclination and health status Health Info Libr J 2011 28 200 209 10.1111/j.1471-1842.2011.00947.x 21831219

[B46] Koo M Krass I Aslani P Enhancing patient education about medicines: factors influencing reading and seeking of written medicine information Health Expect 2006 9 174 187 10.1111/j.1369-7625.2006.00381.x 16677196 PMC5060343

[B47] Levinson W Kao A Kuby A Thisted RA Not all patients want to participate in decision making. A national study of public preferences J Gen Intern Med 2005 20 531 535 10.1111/j.1525-1497.2005.04101.x 15987329 PMC1490136

[B48] Husson O Thong MS Mols F Oerlemans S Kaptein AA van de Poll-Franse LV Illness perceptions in cancer survivors: what is the role of information provision? Psychooncology 2012 22 490 498 22307579 10.1002/pon.3042

